# Static and Fatigue Load Bearing Investigation on Porous Structure Titanium Additively Manufactured Anterior Cervical Cages

**DOI:** 10.1155/2022/6534749

**Published:** 2022-03-21

**Authors:** Mohit Kumar, Vijay Kumar Meena, Suman Singh

**Affiliations:** ^1^Academy of Scientific & Innovative Research (AcSIR), Ghaziabad 201002, India; ^2^Auxein Medical Private Limited, Sonipat 131028, India; ^3^CSIR-Central Scientific Instruments Organisation, Sector 30-C, Chandigarh 160030, India

## Abstract

This study investigates the static and fatigue behavior of porous and conventional anterior cervical cages. Porous structure titanium anterior cervical cages were manufactured using direct selective laser sintering technique. Four different types of cervical cages were designed and manufactured, among which three designs consist of porous structure (type 1, type 2, and type 3) and manufactured using metal 3D printing. Remaining one design (type 4) was manufactured using conventional machining and did not consist any porous structure. All types of manufactured cages were tested in compression under static and fatigue loading conditions as per ASTM F2077 standard. Static and fatigue subsidence testing was performed using ASTM F2267 standard. Static compression testing results of type 1 and type 4 cages reported higher yield load when compared to the type 2 and type 3 cages. Static subsidence testing results reported almost 11% less subsidence rate for additively manufactured cages than the conventional cages. Fatigue subsidence testing results showed that type 2 and type 3 cages can withstood approximately 21% higher number of cycles before subsidence as compare to the type 1 and type 4 cages. During fatigue testing, all the cages design survived 5 million cycles at the 3000 N loading. For 6000 N and 8000 N, loading rate type 2 and type 3 cages showed lower fatigue life when compared to other cages design. Since fatigue life of type 2 and type 3 cage designs were reported lower than other cages design, it is concluded that the performance of the additively manufactured porous cages can be significantly varied based upon the cage design features.

## 1. Introduction

Anterior cervical interbody fusion procedures are the most common and effective methods to treat with the cervical spine pathologies such as degenerative disc diseases, instabilities, and pseudarthrosis or failed spondylosis [1–4]. Cervical spine pathologies may be caused due to the spinal tumors, osteoporosis, and vertebral fractures. Anterior cervical interbody fusion is intended to replace cervical intervertebral discs and to fuse adjacent vertebral bodies at vertebral levels C2–C7 following anterior cervical discectomy for reduction and stabilization of the cervical spine. To obtain the biological fixation, autologous bone or bone graft substitute can be used with the cervical cage. Titanium and PEEK materials have excellent biocompatibility and biomechanical behavior. Anterior cervical cages made of these materials are most commonly used treatment options for cervical interbody fusion. These cervical cages had shown a high percentage of excellent clinical outcomes in the past [5–7].

Although a high clinical success for metals and polymer-based anterior cervical interbody fusion cages have been reported, there are still some studies that reported the complications related to the subsidence and dislocation of cages. Lee et al. reported the subsidence rate of 36.4% and 29.1%, respectively, when 41 patients treated with PEEK material standalone cervical cages [8]. Bartles et al. reported 29.2% subsidence rate during the treatment with cervical carbon fiber PEEK cages [9]. Titanium cervical cages have reported more subsidence effect or screw-related problems than PEEK or carbon fiber PEEK cages [10]. The main reason for the subsidence-related problems for the titanium cages is stress shielding effect, which is caused due to the mismatch of the titanium and bone material young's modulus [11, 12]. There are many experimental and numerical simulation studies that have investigated the subsidence and implant failure due to stress shielding effect [13, 14].

Development of porous structure implants with comparable young's modulus as that of bone is a topic of current interest for researchers. It is well known that porous structured implants promote bone ingrowth and help to reduce the stress shielding effect and increases the osseointegration. Young's modulus of the titanium implants can be reduced using the different pore sizes and pore structures [15]. Various preclinical and clinical studies demonstrated that porous structure implants enhance the bone ingrowth [16, 17]. Vance et al. studied the stress shielding effect using customized additively manufactured implant for tibia bone [18]. Various methods have been reported to manufacture porous structure implants such as electron beam manufacturing, direct metal laser sintering, plasma spray coating, conventional powder metallurgy frontier, and space-holder technique [19, 20]. Additive manufacturing techniques are opening the new possibilities to manufacture those customizable complex designs that have highly interconnected and optimized pores [19, 21, 22]. Various authors have evaluated the mechanical properties of the additive manufactured samples. Han et al. reported the mechanical properties based on the optimized density [23]. Metal 3D printing process parameters such as laser power, scan speed, and hatch spacing play a vital role to have desired mechanical properties. Various authors attempted to report the optimized 3D printing parameters for porous-based structures [24, 25].

Various studies demonstrated the bone ingrowth in animal using metal 3D printed cages [21, 26, 27]. As per authors best knowledge, there is only one published clinical trial study available for metal 3D printed anterior cervical cages [28]. Moreover, Mark et al. suggested the need of conducting long-term studies to demonstrate the safety of 3D printed porous cages. Long-term safety and effectiveness of the 3D printed porous cervical cages are still not clear. Moreover, degradation problems associated with the metal 3D printed implants also have been reported [29]. Biomechanical testing is a key parameter to verify the long-term in vitro performance using static and fatigue loading conditions [22]. There are testing standards available to verify the biomechanical performance of the spinal cages such as ASTM F2267 and ASTM F2077. Compression-based static and fatigue testing techniques are used to evaluate the mechanical properties of the cages. Subsidence-based tests are effective to evaluate the subsidence rate of the cages.

In 2019, Lim et al. had designed a porous titanium and PEEK composite spinal cages with different pore sizes manufactured using additive manufacturing. The authors evaluate the static and fatigue behavior of the composite cages using compression tests, static torsion tests, and subsidence tests. Various researchers attempted to evaluate the fatigue behavior of metal 3D printed porous structure scaffolds that are being designed for implants applications [30–36]. Most of the existing studies have utilized the porous structures scaffold for mechanical testing. To the best of our knowledge, there is no published study available demonstrating the mechanical behavior of the titanium porous structure anterior cervical cages considering the long-term fatigue loading. In this study, we have hypothesized that titanium porous structure cervical cages can have equivalent in vitro performance to the conventional cages. In the study, we attempt to evaluate the in vitro performance of four different anterior cervical cages.

## 2. Materials and Methods

### 2.1. Design and Manufacturing

**Figure 1:**
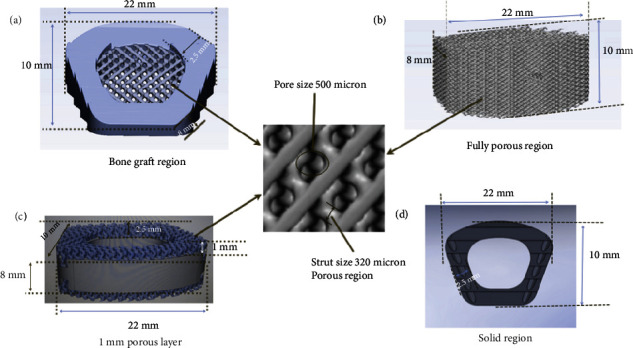
(a) Type 1 cage. (b) Type 2 cage, (c) Type 3 cage. (d) Type 4 cage.

Four types of designs (length 22 mm × width 10 mm × height 8 mm) were manufactured, among which three designs were manufactured using additive manufacturing, and remaining one design was manufactured by conventional machining process. Three different designs of porous structure cages were manufactured. Manufactured cages were divided into the four types (type 1, type 2, type 3, and type 4). A hybrid cage consists of solid and porous region named type 1 was designed. Type 1 cage consist the porous structure at the mid of the cage body. In the type 2 cage, fully porous structure-based cage was designed. In type 3 cage, 1 mm layer of porous structure at the anterior surface of the cage was designed. Type 4 cage consist conventional solid cage design without any porous structure.  Figure 1 represents the dimensions of all designed cervical cages.

It is reported in the literature that porous scaffolds with pore size of 400-600 micron promotes better bone ingrowth [15]. All porous structure-based anterior cervical cages (type 1, type 2, and type 3) were designed with 500 micron pore size with 65% porosity using diamond structure. Simpleware software (Synopsys, USA) was used to design the porous structures.  Figure 1 represents the geometry and dimensions of the pores structure.

**Figure 2:**
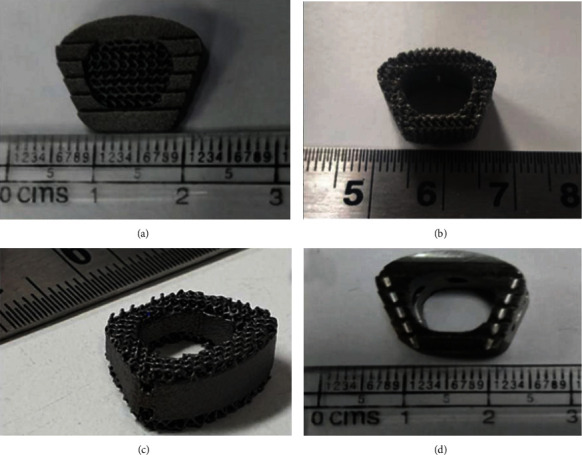
Representative finished specimens from all four types of cages.

All designs (type 1, type 2, and type 3) containing porous structure were printed by using selective laser melting technology-based printer EOS M290, Germany. The metal 3D printing process was conducted with a 400 Watt Yb (Ytterbium) fiber laser, layer thickness of 30 *μ*m, a laser scanning velocity of 400 mm/s, hatch spacing of 70 *μ*m, and a build plate temperature of 35°C. Alternating hatch pattern was used for the scanning strategy. The powder particle size was 30 *μ*m. The manufacturing process was carried out in an argon atmosphere to prevent oxidation. After printing, all the specimens were undergone through heat treatment process for 8 hours at temperature of 800°C. After the heat treatment, shot peening of samples was performed to achieve the optimum surface finishing. Ceramic beads of average grain size 125–250 *μ*m were used during the validated shot peening process [37]. This process was carried out for 2-3 minutes. After the shot peening, ultrasonic cleaning was performed using acetone and water solution. CAD model of conventional anterior cervical cage was provided by Auxien Medical Private Limited, India. Conventional cages were manufactured from the Ti6Al4V ELI. Raw material of titanium was used as per ISO 5832-3 [38, 39]. Mazak Variaxis J 500, Japan milling machine was used for the machining purpose. Type 4 specimen was undergone for ultrasonic cleaning. Heat treatment step was excluded for the type 4 specimen.  Figure 2 represents the manufactured anterior cervical cages.

### 2.2. Pore Size, Surface Roughness, and Young's Modulus Evaluation of Manufactured Samples

**Figure 3:**
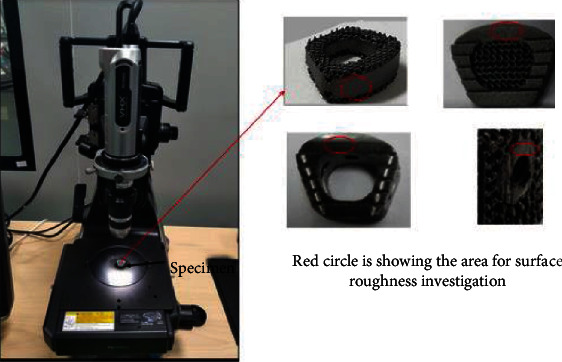
Set up for surface roughness measurement.

All the additively manufactured porous cages and conventional cages were processed through quality control process for dimensional verification. Five specimens from each group were measured for pore size and surface roughness. The surface roughness and pore size values were determined by calculating the average of these measurements. Measurements were recorded using the digital microscope (Keyence VHX series). A custom-developed method was used for measurement.  Figure 3 represents the set up for the surface roughness measurement. The magnification used was 200× and during the surface roughness measurement laser beam was focused on the available solid area of the samples. Surface roughness measurements were taken from 500 × 500 *μ*m length on each specimen. Pore size measurement was carried out at different pore locations of each cage with 200× magnification.

**Figure 4:**
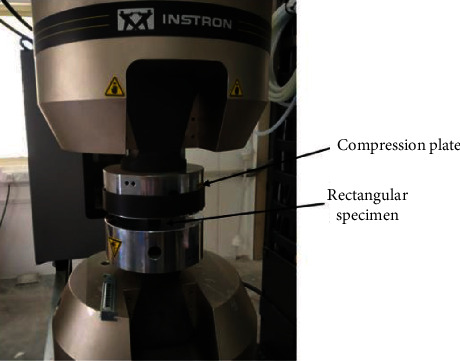
Compression testing setup for Young's modulus evaluation.

For the Young's modulus evaluation, three specially designed porous rectangular blocks (15 mm × 15 mm × 15 mm) were tested. These specimens were designed and printed with 500 micron pore size with 65% porosity using diamond structure, same as the final porous cages design. The static compression testing was carried out as per ISO 13314: 2011 [40]. Instron 100 kN axial servo hydraulic testing system was used to conduct testing and evaluating results such as load vs. displacement, stiffness. Young's modulus was evaluated using the blue hill software.  Figure 4 represents the final test setup for the compression testing.

### 2.3. Mechanical Testing

**Figure 5:**
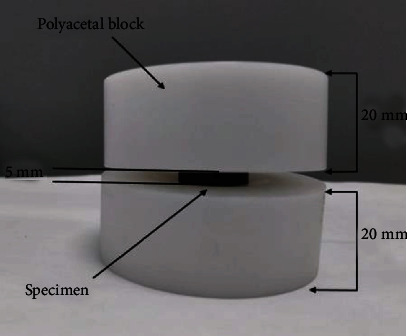
Intradiscal height diagram for fatigue testing.

All four types of cages were tested using various types of loading conditions such as static subsidence loading scenario as per ASTM F2267 guidelines [41], static compression testing, and compression fatigue testing as per ASTM F2077 guidelines [42]. Polyacetal blocks were used as bone substitute material for fatigue loading tests, and stainless steel blocks were used for static loading-based testing.  Figure 5 represents the intradiscal height diagram for each test.

For conventional cages (type 4), polyurethane material of density 0.16 g/cm^3^ was used as the bone graft substitute [43].

#### 2.3.1. Static Testing

**Figure 6:**
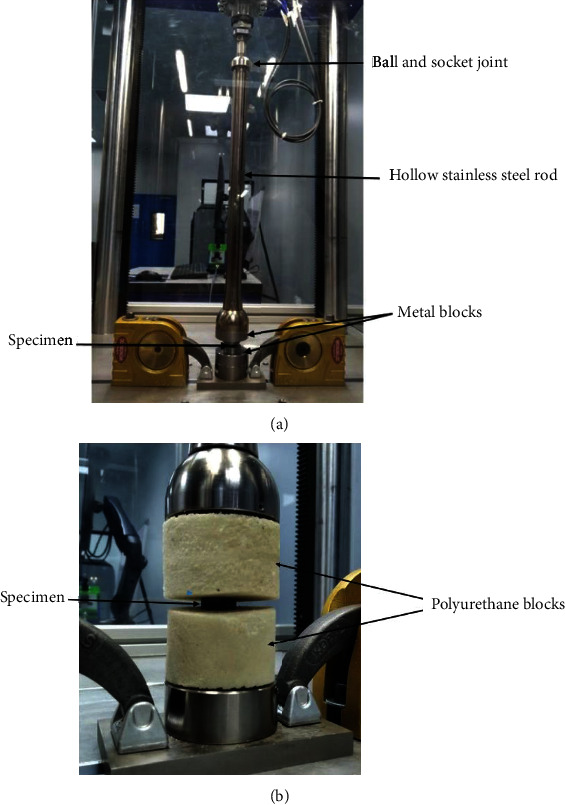
(a) Test set up for static compression testing. (b) Test set up for subsidence static testing.

Static loading based tests were performed using single-station loading fixture mounted on a Dynamess Pneumatic TP 10 with 10 kN load frame (DYNA-MESS Prüfsysteme, Germany). Five specimens for each group were loaded to failure for two static loading modes: static compression loading and static subsidence loading. For static compression test, a load was applied at rate of 5 mm/min with stainless steel hollow push rod for static testing. A preload of 10 N was used for each test.  Figure 6(a) represents the actual testing setup for the static compression testing. For subsidence static testing, polyurethane blocks as specified in ASTM F 1839 (density 160 kg/m^3^, Polynate Foams Private Limited, Bengaluru, India) were used to determine the cage's propensity to subside [44]. Loading was applied at rate of 5 mm/min.  Figure 6(b) represents the actual test set up for the static subsidence testing.

From the static compression test, the load vs. displacement curves were plotted for each of the specimens, however, no permanent failure for type 1 and type 4 cages was recorded. Thus, ultimate compressive force could not be determined. Instead of ultimate force, 0.2% compressive yield force and corresponding displacement were calculated for these specimens. Ultimate compressive force, ultimate compressive displacement and stiffness were calculated for type 2 and type 3 cages. From the static subsidence test, yield subsidence load and corresponding displacements were recorded for all types of cages. Yield subsidence load was defined as the applied load required to cause a permanent deformation equal to the offset displacement. Stiffness of the each test was evaluated by slope ((*Y*_2_ − *Y*_1_)/(*X*_2_ − *X*_1_), where *x* and *y* are load and displacement coordinates) of the initial linear portion of the load-displacement curve.

#### 2.3.2. Fatigue Testing

**Figure 7:**
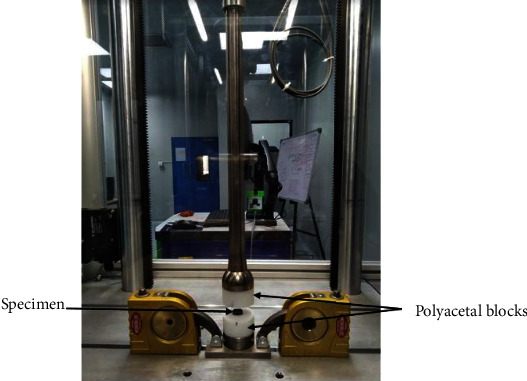
Test set up for fatigue testing.

For the fatigue compression testing, three specimens from each type of cages were loaded to 200-2000 N, 400-4000 N, and 800-8000 N cyclic (sinusoidal wave form) loading, respectively. Sample size of 9 was used for each type of cages. At each loading rate, 3 samples were used for testing. The specimens were placed in between two vertebral body substitutes (polyacetal blocks). For all tests, separate polyacetal blocks were manufactured for each specimen and discarded after testing. An axial preload of 100 N was used for each test.  Figure 7 represents actual test set up for the compression fatigue testing. Data recording for S-N curve was performed by Dyna-Tcc software. Specimens were cyclically loaded to fatigue failure or run out cycles (5 million) at 10 Hz loading frequency. R ratio of 10 was used for each test. For the weight reduction measurement, initial and final weights for each specimen were recorded using the micro balance instrument (Mettler Toledo, USA, XPE 56). Weight reduction was calculated by subtracting initial and final weight.

For the fatigue based subsidence test, the specimens were impacted into the space in between two polyurethane foam blocks (density 160 kg/m^3^). This grade of PU foam is commonly used as the representative of the osteoporosis bone substitute material. Based on the static subsidence results, a 4 mm of axial displacement was considered as the subsidence failure. All the specimens were loaded to 10-100 N, 20-200 N, and 30-300 N loading ranges up to 4 mm axial displacement. Load vs. number of cycle curves, maximum displacement, and failure mode were recorded for each specimen.

#### 2.3.3. Statistical Analysis

Statistical analysis was performed by Minitab 16. Statistical significance of difference for various variables such as yield load, subsidence rate, and reduction in weight was calculated using ANOVA (one-way analysis of variance). Static testing data were reported as mean ± standard deviation, and statistical significance was considered at *p* < 0.05.

## 3. Results

### 3.1. Pore Size, Surface Roughness, and Young's Modulus Measurement

**Figure 8:**
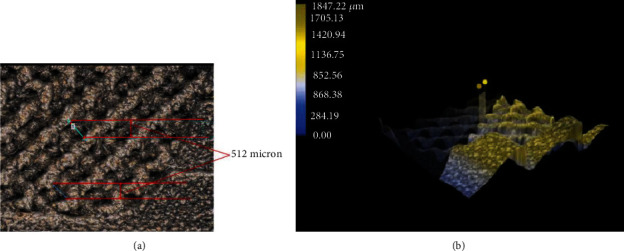
(a) Test set up for measurement using microscope at 200×. (b) Pore size measurement using surface topology.

The mean pore size of the type 1, type 2, and type 3 specimens (*n* = 15) was 511 *μ*m (±2.3 *μ*m), 512 *μ*m (±1.9 *μ*m), and 510 *μ*m (±5.4 *μ*m), respectively. Conventional cages group (type 4) had better surface finish than all other types of cages. Surface roughness measured for type 1, type 2, type 3, and type 4 is presented in Table 1. Figures 8(a) and 8(b) represent the pore size measurement at 200×.

**Figure 9:**
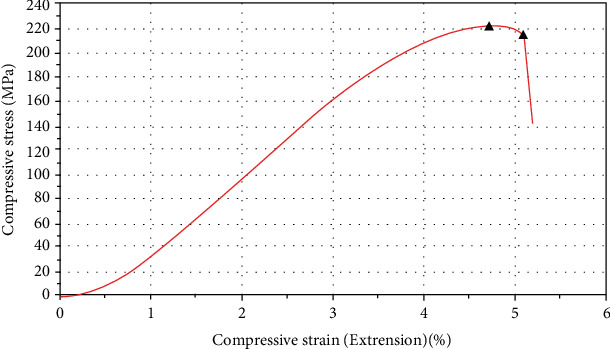
Representative stress vs. strain curve for Young's modulus evaluation testing.

The reported average Young's modulus was 6773.6 MPa (±266.9 MPa), and the average compressive strength was 276.6 MPa (±19.5 MPa). Mean maximum force at break was 47003.8 N (±1657.6 N), and corresponding mean displacement was 0.79 mm (±0.03 mm).  Figure 9 represents the load vs. displacement curve.

### 3.2. Static Testing Results

#### 3.2.1. Static Compression Test

**Figure 10:**
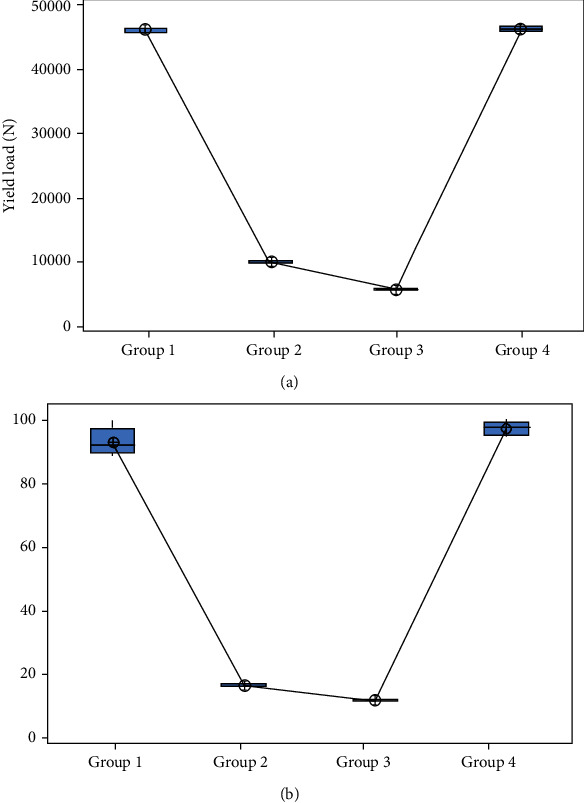
(a) Yield load comparison using box and whisker diagram (*p* value is < 0.05). (b) Stiffness comparison using box and whisker diagram (*p* value is < 0.05).

The structural properties of the type 1, type 2, and type 3 cages were significantly affected by the cage design. Specifically, layer-based porous cage design (type 3) had significantly lower yield force than other cages design. Conventional cage design (type 4) had higher yield force value than all other designs. Mean yield load or failure load recorded for type 1, type 2, type 3, and type 4 was 46155 N (±298.6 N), 10007.8 N (±220.4 N), 5656.4 N (±206.1 N), and 46349 N (±405.3 N), respectively. There was a significant difference (*p* < 0.05) between the stiffness values of the all four types of cages. Type 1 and type 4 had approximately similar values of stiffness. Type 3 specimen had significantly lower stiffness value than all other types of cages. Mean stiffness recorded for type 1, type 2, type 3, and type 4 was 93.2 N/m (±4.44 N), 16.36 N/m (±0.544 N/m), 11.68 N/m (±0.383 N/m), and 97.64 N/m (±2.133 N/m), respectively.  Figure 10(a) represents the compression yield load comparison, and Figure 10(b) represents the stiffness comparison of all types of cages using box and whisker diagram.

**Figure 11:**
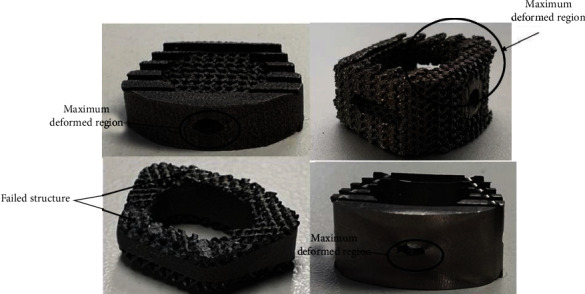
Representative picture of failure mode for all types of cages.

Moreover, for type 2 and type 3 cages, a brittle failure was recorded and small broken wear debris particles (1 micron to 100 micron) from the porous structure were observed after testing. No sign of debris particles for type 1 and type 4 cages design was observed, only permanent deformation was recorded.  Figure 11 represents the failure modes for all the group's specimens.

#### 3.2.2. Static Subsidence Testing

**Figure 12:**
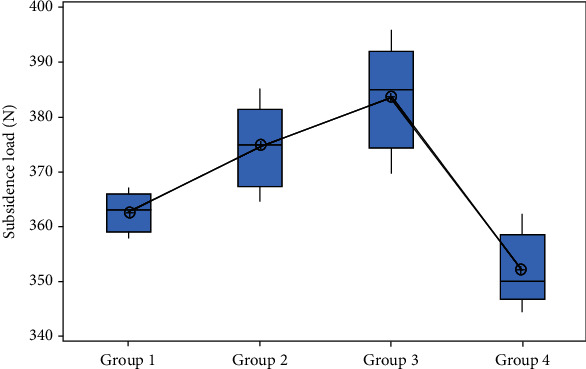
Subsidence load comparison using box and whisker diagram (*p* value is < 0.05).

For these tests, subsidence rate was also significantly affected by the design features. Type 1 cage design had significantly lower subsidence yield load than other types of cages. Conventional cage design (type 4) had significantly higher subsidence yield load than other types of cages. Mean yield subsidence load recorded for type 1, type 2, type 3, and type 4 was 362.6 N (±3.65 N), 374.6 N (±7.64 N), 383.6 N (±9.58 N), and 352 N (±6.67 N), respectively, and corresponding subsidence displacement recorded was 3.77 mm (±0.17 mm), 4.19 mm (±0.07 mm), 4.43 mm (±0.08 mm), and 4.06 mm (±0.12 mm).  Figure 12 represents the yield subsidence loads comparison of all groups using whisker plot diagram.

### 3.3. Fatigue Testing Results

**Figure 13:**
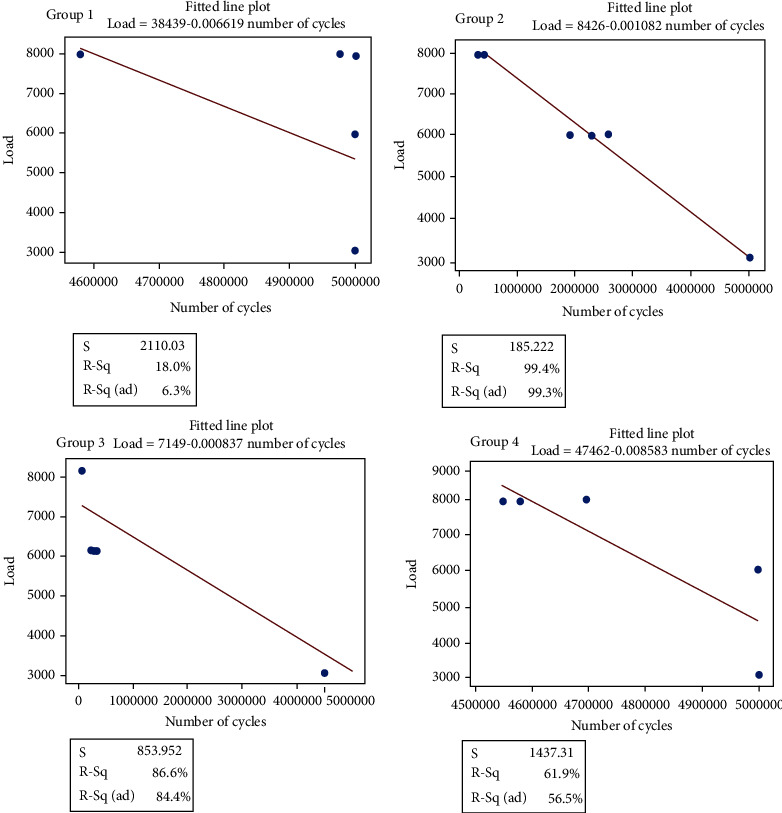
S-N curve plotted using fitted plot line for each type of cages.

During compression dynamic testing, specimens were loaded to 3000 N, 6000 N, and 8000 N compressive load. During these loading conditions, type 1 and type 4 cages survived the 5 million cycle limit without any visible failure. No sign of debris was observed after the testing. A constant displacement was recorded throughout the testing. Type 2 and type 3 specimens were survived 5 million loading cycles only for 3000 N loading but the sign of debris was observed after completion of testing. At the beginning of testing (up to 5000 loading cycles), significant changes in the displacement were recorded. For type 2 and type 3 specimens, significant reduction in the weight of specimens was recorded. During 6000 N loading range, type 2 and type 3 cages reported failure at average 2235637 cycles (±315099) and 411186 cycles (±49369.4 cycles), respectively. During 8000 N loading range, type 2 and type 3 cages reported failure at average 250454 cycles (±53024 cycles) and 62771 cycles (±17236 cycles), respectively.  Figure 13 represents the S-N curve fitted by regression analysis for the all types of cages.

For the weight reduction measurement, initial weight and the weight after testing for each specimen were recorded using the microbalance instrument (Mettler Toledo, USA, XPE 56). Weight reduction was calculated by subtracting initial weight and after testing weight.

Moreover, for type 2 and type 3 cages, a significant reduction in the weight of the specimens was recorded. Signs of debris particles were visually observed after the testing. Maximum 0.094721 grams weight reduction for type 3 specimen was recorded. Type 3 specimen reported higher weight loss rate than other types of cages. But the rate of change was not significant. Description of failure mode for each type of cages design is presented in Table 2.

**Figure 14:**
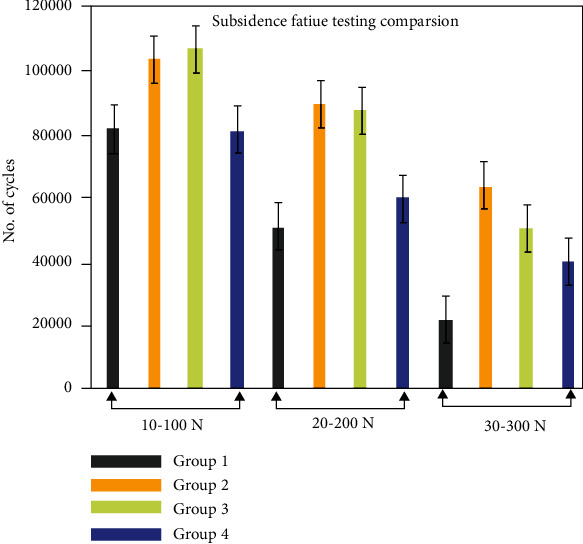
Fatigue subsidence failure comparison between all four types of cages when tested up to 4 mm axial displacement.

Cage design and porous structures have significant effect on the subsidence rate. Fatigue subsidence testing results reported that type 2 and type 3 have higher number of cycles for subsidence with respect to other types of cages. A number of cycles for subsidence at 10-100 N loading range were reported as 8242 (±823) cycles, 10263(±957) cycles, 10671(±763) cycles, and 8156 (±657) cycles for type 1, type 2, type 3, and type 4 cages, respectively. During the 20-200 N loading range, subsidence was reported as 5051 (±512) cycles, 8902 (±467) cycles, 8868 (±414) cycles, and 6000 (±433) cycles, respectively. Similarly, for 30-300 N loading range, subsidence rate was reported as 2131(±389) cycles, 6421 (±311) cycles, 5055 (±467) cycles, and 4000 (±488) cycles, respectively. Type 4 and type 1 cages had achieved subsidence displacement (4 mm) at lower cycles than type 2 and type 3 cages.  Figure 14 represents the subsidence fatigue testing results comparison for all four groups.

## 4. Discussion

The aforementioned results based on the various ASTM tests demonstrate the equivalent performance of type 1 and type 4 cages. From the results, it is clear that the additively manufactured hybrid porous cages (type 1) can behave same as the conventional cages (type 4). Type 1 and type 4 cages reported almost five times more compressive strength than the type 2 and type 3 cages. However, during activities of daily living life, the anterior cervical vertebrae does not experience such a high load. Compressive load limits of 3340 to 4450 N have been calculated for the cervical spine vertebrae by various authors [45, 46]. For all the types of cages, the value of yield load evaluated in the present study is higher than the maximum compressive load of anterior cervical spine. In 2017, U.S. Food and Drug Administration published a systematic analysis of mechanical testing data based on ASTM F2077. Median device yield strength reported was 10,117 N for static axial compression tests [47]. Static compression testing results for type 2 and type 3 cages are consistent with USFDA published results. For type 1 and type 4, compression testing results presented in this study are almost four times higher than USFDA reported results. Despite of the routine application and recommended use of ASTM F2077 for Intervertebral Body Fusion device guidance, there are no recommended values for the yield strength and other structural parameters.

Additively manufactured cages (type 1, type 2, and type 3) showed the lower subsidence rate than the conventional cages (type 4). These cages have almost 11% less subsidence rate than the conventional cages. Shu et al. reported the subsidence load 368.2 to 426.6 N for various titanium spinal cages. These results are consistent with the published literatures [46, 48]. Fatigue subsidence testing results showed that type 2 and type 3 cages can withstand approximately 21% higher number of cycles before subsidence as compare to the type 1 and type 4 cages. Based on the present study results, it was found that additively manufactured cages can significantly reduce the subsidence-related problems of anterior cervical region of the spine.

In the present study, no fatigue failure was observed on the type 1 and type 4 cages. Fatigue testing was conducted on the single station 10 kN load cell. A loading range of 200-2000 N, 400-4000 N, and 800-8000 N were applied on the all types of cages. These load ranges were sufficient to evaluate the S-N curve for all the types of cages, as 3340 N to 4450 N compressive load could be maximum load-bearing capacity of a normal adult's cervical vertebrae, however, even applying a loading range of 800 N to 8000 N load failure could not be determined for the type 1 and type 4 cages. For type 1 and type 4 cages, all the specimens withstood 5 million loading cycles without any failure, evidence of failures existed on the polyacetal blocks. Hairline fractures were observed on the polyacetal blocks. These fatigue results are consistent with the other marketed spinal cages [21]. Based on the compression fatigue study results, it was found that the porous design can significantly reduce the fatigue life of the anterior cervical cages. Type 2 and type 3 specimens were unable to survive 5 million loading cycles when tested beyond 3000 N loading. A significant amount of weight loss reduction was recorded for the additively manufactured cages (type 2 and type 3). This is a key finding of this study. This kind of degradation of the debris particles can lead to a serious adverse event to the patient.

For compression based fatigue testing, recommended maximum force for initial dynamic tests are 25, 50, and 75% of the ultimate static force [42]. Due to the load cell limitation, we were unable to apply the loads more than 10 kN. Subsidence based fatigue testing results also provide the insight into the less subsidence rate for the additively manufactured porous cages. During the fatigue testing, several difficulties were encountered throughout testing, highlighting aspects of the standard that might need improvement, while applying load more than 8000 N the machine generates immense vibration so the tests were stopped immediately. This may be due to the long construct of test set up, thus, there may be an opportunity to improve the testing method so that vibration issues can be controlled.

Surface roughness of the additively manufactured cages can have significance on the mechanical and biological properties. It is reported in the literature that higher surface roughness can lead to lower mechanical properties [49, 50]. Surface roughness also has direct correlation with the osseointegration. Schwarz et al. used calcium phosphate coating on titanium implant and reported that higher surface roughness of Ra 28 *μ*m can have better bone growth when compared to the lower surface roughness values [51]. In the present study, the authors attempted to maintain the surface roughness values from 11 to 16 *μ*m (Ra), which is consistent with the recommended surface roughness values for orthopedic implants applications. In the future, more studies are needed to establish an optimum relationship between surface finishing, osseointegration, and fatigue properties of titanium 3D printed implants.

There are few limitations of the present study, and the shear and torsional based mechanical testing were not performed. Wear tribology studies using spine simulator devices would be helpful to determine the fatigue life and wear debris assessment of the porous cages. Cadaver-based mechanical testing set up would be useful to have more accurate results. In this study, we have used only three types of additively porous cages, and more design should be explored based on the topology optimization.

## 5. Conclusions

In the current study, static and fatigue behavior of additively manufactured porous cages were evaluated. The outcomes of the study are as follows:

- In vitro performance of the additively manufactured porous cages can be affected by the design features
- Additively manufactured porous cages have abilities to reduce the stress shielding effect and may have higher clinical success
- Design optimization of the additively manufactured cages is very important and can have significant effect on the fatigue life. Cervical cages with fully porous design (type 2) and layer-based design (type 3) can reduce the subsidence rate. Thus, the fatigue life reduction and wear debris for such designs can lead to the other serious adverse events
- Hybrid cages design such as type 1 cage can be better solution for the anterior cervical fusion surgery since the static and fatigue load bearing performance of these cages are equivalent to the conventional cages

## Figures and Tables

**Table 1:** Surface roughness measurement for each group.

Surface roughness		
	Ra (*μ*m)	Rz (*μ*m)
Type 1	11.9 (±0.70)	31.3 (±0.97)
Type 2	16.6 (±1.5)	34.7 (±1.16)
Type 3	12.73 (±1.3)	31.4 (±1.3)
Type 4	6.8 (±0.99)	9 (±0.70)

**Table 2:** Description of weight loss reduction after fatigue testing.

Average weight loss for (*n* = 3)	Loading range			Description of failure mode
	3000 N	6000 N	8000 N	
Type 1	0.013612 gram	0.0145612 gram	0.014451 gram	No fatigue failure for cages for all loadings. For 8000 N loading fracture observed on polyacetal blocks
Type 2	0.094123 gram	0.093576 gram	0.095343 gram	At 3000 N loading specimens withstood 5 M cycles, at 6000 N and 8000 N loading fatigue failure/maximum displacement recorded
Type 3	0.9125160 gram	0.092312 gram	0.094721 gram	At 3000 N loading specimens withstood 5 M cycles, at 6000 N and 8000 N loading fatigue failure recorded
Type 4	0.011423 gram	0.0145512 gram	0.012451 gram	No fatigue failure for cages for all loadings. For 8000 N loading fracture observed on polyacetal blocks

## Data Availability

The data supporting the results can be found at the reference section of the manuscript.
